# Peri-Implant Tissue Adaptation after Implant Rehabilitation with Shoulderless Abutments with 24 Months of Follow-Up

**DOI:** 10.1155/2021/6689446

**Published:** 2021-01-07

**Authors:** Luca Casula, Alex Gillone, Davide Musu

**Affiliations:** ^1^Oral Surgery Resident, Vita Salute University, Milan, Italy; ^2^Division Director of Periodontology, East Carolina University, Greenville, USA; ^3^Department of Endodontology, Academic Centre for Dentistry Amsterdam (ACTA), Amsterdam, Netherlands

## Abstract

An 11-year-old girl presented with agenesis of the maxillary lateral incisors. Orthodontic treatment was performed to close the midline diastema and create adequate space between the central incisors and canines to replace the missing maxillary lateral incisors on both sides. Two-piece implants were placed, and shoulderless abutments were prepared following the “biologically oriented preparation technique” (BOPT) protocol. The soft tissues were allowed to heal directly on the prosthetic emergence profile of the interim crown restorations after implant exposure. Two months later, the gingival tissue adapted to the prosthetic components in a specular manner. No complications were noted at 24 months. The BOPT protocol, originally described for natural teeth, may be applied to dental implants with shoulderless abutments.

## 1. Introduction

Implant abutment morphology influences marginal bone loss and the biologic width of the peri-implant mucosa [1–5]. Souza et al. [4] reported that an implant abutment design with a wide and more divergent emergence profile induces an apical displacement of the peri-implant biologic width and an increased bone loss compared to an implant abutment with a narrower emergence profile. Agustín-Panadero et al. compared implants with divergent transmucosal collars to those with a convergent collar design and observed that the latter resulted in decreased bone loss [5]. A smaller abutment diameter, relative to the implant platform, seems to reduce the postrestorative crestal bone remodeling, which results in greater bone preservation [6, 7]. However, the role of the abutment design in marginal bone loss and peri-implant soft tissue stability is currently unknown [8, 9]. The crown is usually adapted against the horizontal finish line of the implant-supported cement-retained prosthesis, and together, they form the emergence profile of the implant-supported restoration [10]. The emergence profile of the implant-supported restoration is determined by the contour of the crown as it relates to the adjacent tissues [11]. Similar to natural abutment teeth [12–14], a shoulderless implant abutment has a vertical area without a finish line where the crown margin is placed [15]. The absence of a defined horizontal finish line allows the interim restoration to move in an apical or coronal direction, allowing the tissue to adapt to the emergence profile of the crown restoration in a specular manner [12]. This concept, which was introduced with the biologically oriented preparation technique (BOPT) protocol [12–14], allows for the thickening of the soft tissues in the coronal direction and results in excellent esthetics with both the implants [16–19] and natural teeth [20, 21]. The use of the “adaptation forms and profiles concept” [12] in rehabilitative treatment involving natural teeth [12–14, 20, 21] and implants with shoulderless abutments [16–19] has been well documented; however, the adaptation of the gingiva to the emergence profile of prosthetic crowns has not been reported for the implant-supported cement-retained restorations methodically. This clinical case report describes a patient with agenesis of the maxillary lateral incisors. Orthodontic treatment was performed to create space for implant placement. Two implant-supported cement-retained prostheses were provided, using a protocol similar to the BOPT for natural teeth [12]. The step-by-step gingival movement toward the crown emergence profile during tissue maturation is described up to two years after crown delivery.

## 2. Case Report

**Figure 1:**
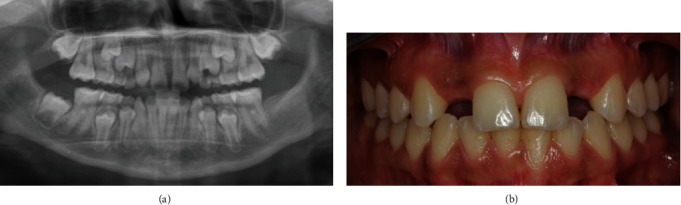
(a) Panoramic radiograph showing agenesis of both maxillary lateral incisors. (b) Facial view at the end of the orthodontic treatment showing the space maintained with the removable orthodontic retainer.

An 11-year-old girl was referred to the Department of Dentistry at the San Raffaele Hospital in Milan, Italy. The patient expressed dissatisfaction with smile, due to misaligned teeth and spaces between the anterior teeth. Radiographic examination revealed the agenesis of the maxillary lateral incisors (Figure 1(a)). A multidisciplinary treatment plan was formulated, which included orthodontic treatment for the midline diastema closure, focusing on creating adequate space between the central incisors and the canines to replace the missing maxillary lateral incisors on both sides. Two implant-supported cement-retained prostheses were planned at the end of the peak of adolescent growth for replacing the lateral incisors [22].

After orthodontic treatment, the patient wore a removable retainer to maintain the space gained for replacement of the lateral incisors with implants (Figure 1(b)). The maxillary implant surgery was performed with the aid of cone beam computed tomography (CBCT) when the patient was 19 years old. Two 3.3 mm diameter implants (Win-Six Biosafin, Italy) were placed in the area of the lateral incisors, with the implant platform positioned at the buccal crest level and with an insertion torque of 30 Ncm.

**Figure 2:**
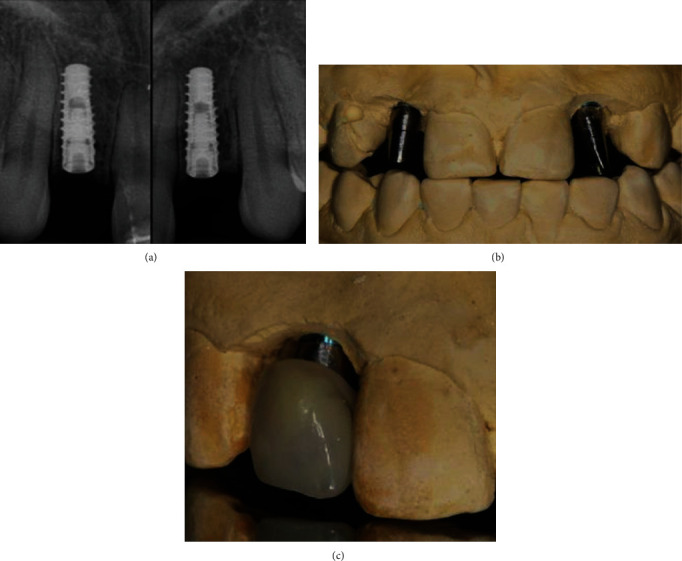
(a) Implants on lateral incisors positioned 4 months after placement. (b) The shoulderless abutments directly adjusted on the master cast. (c) The crown was placed 3 mm away from the analog (3 mm from the bone crest).

Immediate loading was avoided, and the patient wore a removable retainer during osseointegration of the implant [23] . Four months after implant placement, two periapical radiographs were taken confirming that the implant platforms were at the level of the bone crest (Figure 2(a)). After confirmation, we proceeded to the second stage of maxillary surgery. First, the two alginate impressions were made, and wax interocclusal records in maximal intercuspation were taken and sent to the dental laboratory for fabrication of the interim complete crown restorations. The gingival thickness at the implant site was determined to be 4 mm by inserting a K-file with an endodontic stop at the center of the edentulous ridges. Subsequently, the implants were exposed by raising a minimal full-thickness flap and moving the occlusal keratinized tissue to the buccal aspect.

**Figure 3:**
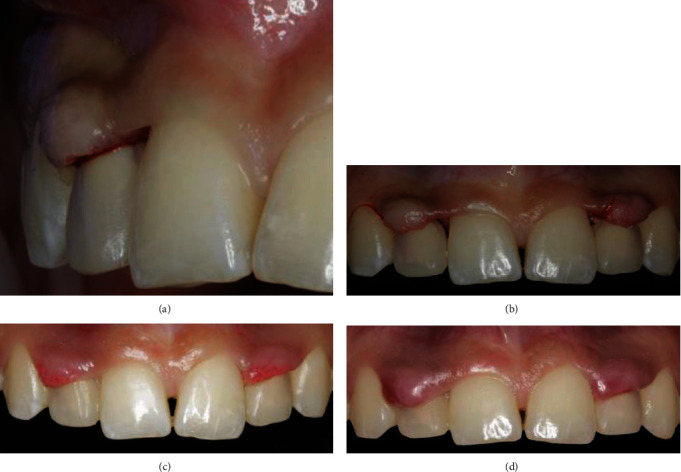
(a) The keratinized tissue moved from the occlusal side and was sustained by the augmented emergence profile of the crown. (b) Peri-implant mucosa formation and gingival adaptation along with the emergence profile of the crown restoration: inflammatory phase with the soft tissues sustained by the immediately placed crowns. (c) Soft tissues at four days of follow-up with minimal inflammation. (d) Soft tissues appeared inflamed and elongated in a more coronal position at one week of follow-up.

The impression copings (Win-Six Biosafin, Italy) were connected to the implants, and an impression was made with vinyl polysiloxane material (Putty and Light Elite HD, Zhermack, Italy), using a perforated custom tray. Two implant analogs were connected to the copings inside the impression, and the cast was immediately poured using the type 4 dental stone. Two modifiable cylindrical shoulderless implant abutments (MF, Win-Six Biosafin, Italy) were connected to the analogs and adjusted (prepared without a finish line) directly on the cast to achieve the appropriate inclination for placement of the temporary crowns. The shoulderless abutments were prepared in the same manner as the natural abutment teeth in the BOPT protocol [12–14] (Figure 2(b)), and the interim complete crown restorations were directly relined over the implant abutments on the implant cast, resulting in an augmented emergence profile (Figure 2(c)) [12]. The interim restorations were placed 3 mm coronally from the analogs that corresponded to the implant platforms (bone crest level), based on the previously measured gingival thickness (4 mm). This ensured that the adequate space remained for biologic width formation (Figure 2(c)) [24]. The temporary abutments were fixed to the implants, and the interim restorations were cemented using a temporary cement (Temp Bond, Kerr, USA). The keratinized mucosa taken from the occlusal space was supported by the overcontoured emergence profile [11] of the crown during the healing process on the buccal aspect (Figure 3).

**Figure 4:**
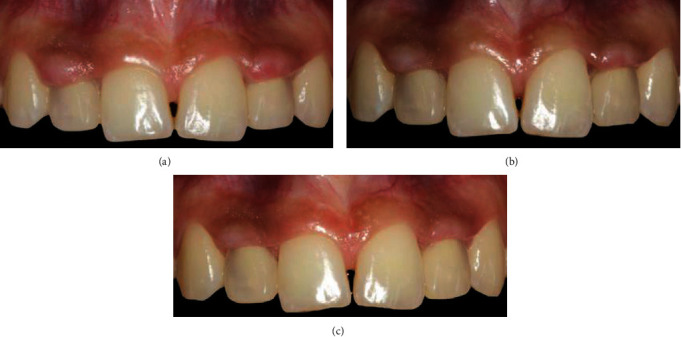
Peri-implant mucosa formation and gingival adaptation along with the emergence profile of the crown restoration: adaptation phase. (a) Soft tissues at two weeks of follow-up receded in an apical position and appeared without inflammation. (b) Soft tissues at 1 month of follow-up were adapted along the emergence profile of the crowns. (c) Soft tissues at 2 months of follow-up were stable, and the interdental spaces were completely filled by the interdental papillae.

**Figure 5:**
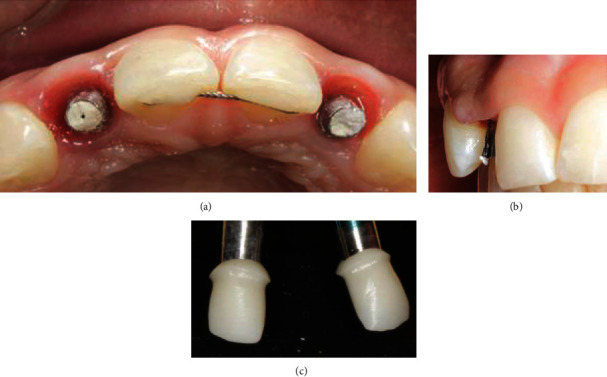
(a) Occlusal view of the peri-implant mucosa 2 months after interim restoration placement. (b) Lateral view showing that the keratinized gingiva adapted and conformed to the prosthetic emergence profile of the interim restoration in a specular way. (c) The definitive restoration frameworks with the reinforced collars that allowed a thick layer of ceramic at the margin of the crown.

After the interim restoration placement, the patient returned to the dental department periodically for 2 months during the healing process, which was documented using photographs. Four days after interim restoration placement, the keratinized tissue appeared inflamed and was in a more coronal position (Figure 3(c)) than before (Figure 3(b)). During the one week follow-up, the keratinized tissue appeared swollen, reddened, and more coronally elongated compared to that during the previous appointment (Figure 3(d)). During the second week of follow-up, the redness and swelling disappeared, and the keratinized tissue appeared to recede more apically (Figure 4(a)). At the 1-month follow-up, the gingival tissue appeared healthy and had adapted to the prosthetic emergence profile of the interim restoration in a specular manner (Figure 4(b)). At the 2-month follow-up, the interdental spaces were completely filled by the interdental papillae, and the crown margins were located 1 mm subgingivally (Figure 4(c)). The tissue appeared pink and healthy, even in the transmucosal area. On the occlusal view, the rounded profile of the gingiva was appreciable and specular to the emergence profile [11] of the interim crowns (Figure 5(a)). The adaptation of the tissue against the overcontoured emergence profile of the crowns was clearly appreciable on the lateral view (Figure 5(b)). After the previously described 2 months of tissue maturation, the patient visited the department for final impressions. The temporary restorations and analogs were removed, and the height of the transmucosal soft tissue was determined, with a dental probe placed between the implant platform and the gingival margin, to be 4 mm for both implants. The bone impression copings (Win-Six Biosafin, Italy) were connected to the implants, and an impression was made with vinyl polysiloxane material (Putty and Light Elite HD, Zhermack, Italy) using a perforated custom tray. An alginate impression of the mandibular arch and interocclusal record in maximal intercuspation were obtained and sent to the dental laboratory for the fabrication of two definitive implant abutments with a single zirconia framework for each. The dental technician was instructed to prepare wax patterns and cast customized titanium abutments without a finish line. The dental technician was also instructed to place the margins of the final crowns 1 mm subgingivally, according to the conditioned soft tissue during the interim stage. A reinforced collar was fabricated by the dental technician, to strengthen the feather-edged margin (Figure 5(c)) [10]. Thus, the ceramic layering of the framework was performed along the reinforced collar of the chamfer, to prevent the fabrication of a crown with a thin ceramic margin.

At the following clinical visit, the abutments and zirconia frameworks were evaluated, and new interocclusal wax in maximal intercuspation was made to check the occlusion again. The accuracy of the framework's fit was confirmed clinically with dual-cured paste (Fit Checker Advanced, Gc Corporation, Japan). A framework transfer impression with polysiloxane material (Putty and Light Elite HD, Zhermack, Italy) was made, to capture the framework-soft tissue relationship. The impression was sent to the dental technician with a request that the final restorations be shaped in a manner that allowed the emergence profile to sustain the mature soft tissues. The final restorations were clinically checked and sent for the final glaze. At the final appointment, the finished crowns were cemented with temporary cement (Temp Bond, Kerr, USA), and the patient expressed satisfaction with the esthetics.

**Figure 6:**
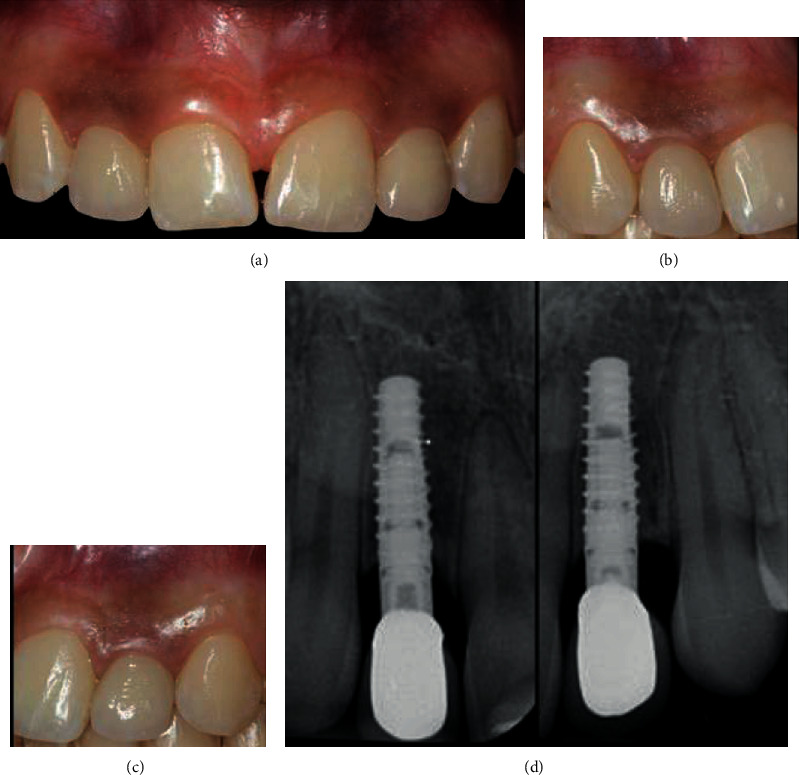
(a) Facial view of the definitive restorations at the 24-month follow-up. (b) Right lateral view of the definitive restorations at the 24-month follow-up. (c) Left lateral view of the definitive restorations at the 24-month follow-up. (d) Periapical radiographs at the 24-month follow-up.

At the 24-month follow-up visit, no biological or technical complications were noted, and the gingiva appeared thick, pink, healthy, and completely adapted to the definitive restoration (Figure 6). The papillary filling was achieved in the interdental spaces. At the 24-month follow-up, minimal bone remodeling of approximately 0.5 mm was detected, which was attributed to abutment installation and initial loading (Figure 6) [25].

## 3. Discussion

The healthy coexistence of dental restorations and the peri-implant structures is essential and is the primary goal of a prosthodontist. The peri-implant tissues should not bleed on probing, and a tight seal should exist between the peri-implant mucosa and the transmucosal component [25]. The abutment design for natural teeth and implants presents a well-defined horizontal finish line or a vertical area without a finish line for the crown margin [10, 26]. A vertical preparation without a finish line in natural abutment teeth [12] results in the formation of an overcontoured cervical portion of the prosthetic crown [27] and has been suspected to have serious implications for the periodontal health of the supporting tissues [28]. The BOPT protocol includes a prosthetic subgingival preparation of the natural abutment teeth without a finish line [12–14] and ensures a good periodontal condition [20, 21]. The “adaptation forms and profiles concept” [12] applied to the natural abutment teeth treated with BOPT [12–14] is applicable for implant-supported cement-retained restorations [11] if shoulderless abutments are used [16–19]. In this case report, the interim restorations were shaped with an augmented contour [12–14] and placed on the shoulderless abutments immediately after flap elevation, while waiting for peri-implant mucosa formation around the restorative components (Figure 3). The positions of the prosthetic crowns and their distance from the bone crest (4 mm at both sites) were clinically determined based on the thickness of the keratinized gingiva, measured at the center of the edentulous ridges before flap elevation. In the scientific literature, the peri-implant mucosal height is reported to be approximately 3 to 4 mm [29], and minimal bone remodeling is expected after the initial loading [25]. Therefore, we placed the interim restorations 3 mm from the bone crest, leaving adequate space for the peri-implant biologic width [23, 29, 30] (Figure 3(c)). Two months after implant loading, the peri-implant mucosa appeared pink, healthy, and completely adapted to the emergence profile [11] of the interim restorations. The healing stages of the peri-implant mucosa documented in this case report are in accordance with the healing stages in animal studies [29]. The first week was characterized by low-grade inflammation of the peri-implant mucosa, which healed and stabilized at the 1-month follow-up [30] and reached complete maturation with a pink and healthy appearance at the 2-month follow-up [29, 30]. The influence of abutment morphology on peri-implant mucosa formation has not been properly elucidated [8, 9, 29, 31], and currently, there are no studies comparing the implant abutments with a finish line with the shoulderless abutments. Nevertheless, the horizontal finish line in implant abutments acts as a vertical stop for seating the crown [10]. In shoulderless abutments, the crown restoration may instead be moved in an apical or coronal direction without invading the biologic width [12–19]. Therefore, the emergence profile and the crown margin can be modified until healthy and stable soft tissues are achieved if the inflammation occurs during the interim stage [16–19].

The BOPT approach used for implant rehabilitation in this study demonstrated a good clinical outcome in the esthetic zone at the 24-month follow-up, with healthy peri-implant mucosa, pink and stippled gingiva, and interdental spaces completely filled by the interdental papillae. The interim crowns were not positioned according to the healed soft tissue, but the soft tissue was allowed to adapt itself against the prosthetic components during the healing stage. The position of the crown was predetermined and was based on the gingival tissue thickness and biologic width dimension. A limitation is apparent in cases in which the gingival thickness is not sufficient to ensure adequate space for the biologic width. Moreover, the technique is time consuming because the interim abutments must be adjusted by the dentist during the second stage surgery, which requires ample experience. The technique has not been backed by scientific literature, and long-term clinical studies are still required to understand the role of the abutment design in peri-implant mucosa formation and to better evaluate, both clinically and scientifically, the outcomes of shoulderless implant abutments.

## Data Availability

The data that support the findings of this study are available from the corresponding author upon reasonable request.
